# Identifying prognostic targets in metastatic prostate cancer beyond AR

**DOI:** 10.1002/2211-5463.70059

**Published:** 2025-05-22

**Authors:** Emily Feng, Eric Feng, Tracy Berg, Isabella S. Nguyen, Lilac G. Nguyen, William Chen, Meng Zhang, David Quigley, Marina Sharifi, Haolong Li, Ilsa Coleman, Peter S. Nelson, Martin Sjöström, Shuang G. Zhao

**Affiliations:** ^1^ Department of Radiation Oncology University of California San Francisco CA USA; ^2^ Department of Human Oncology University of Wisconsin Madison WI USA; ^3^ Department of Medicine University of Wisconsin Madison WI USA; ^4^ Human Biology Division Fred Hutchinson Cancer Center Seattle WA USA; ^5^ Lund University Sweden

**Keywords:** clinicogenomic, DepMap, prostate cancer

## Abstract

Genome‐wide screens using CRISPR/RNAi can identify new therapeutic vulnerabilities in prostate cancer. In this study, we combine DepMap functional screen data with a large gene expression database (*N* = 1012) and clinical outcomes to identify potentially druggable targets. Eight genes (*CYC*, *CYP51A1*, *DHFR*, *EBP*, *KIF15*, *PPM1D*, *SQLE*, and *UMPS*) demonstrated strong dependency in cell lines and were also associated with worse prognosis clinically, representing potential therapeutic targets in metastatic prostate cancer. Four of these (*DHFR*, *EBP*, *KIF15*, and *PPM1D*) demonstrated higher expression in neuroendocrine prostate cancer. Furthermore, all but one (*KIF15*) were not significantly decreased from pretreatment to posttreatment, suggesting that they may remain targetable postabiraterone therapy. All eight genes showed evidence of protein expression in prostate cancers or cell lines. These potentially druggable targets associated with prostate cancer cell line dependency and worse clinical outcomes have also demonstrated literature support as potential targets, supporting further research into their potential clinical relevance as therapeutic targets in prostate cancer.

## Graphical abstract

Genome‐wide functional screens combined with a large gene expression database and clinical outcomes can identify new therapeutic vulnerabilities in prostate cancer. Eight potentially druggable targets demonstrated strong dependency in cell lines, were associated with worse prognosis clinically, and showed evidence of protein expression in prostate cancers or cell lines, representing potential therapeutic targets.

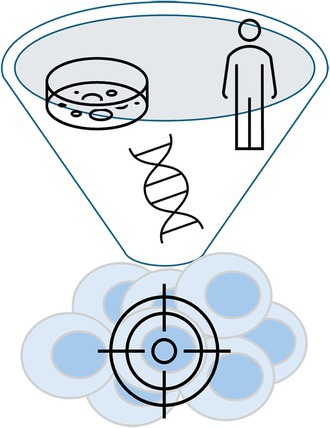

**Abbreviations**
CCLE: cancer cell line encyclopedia
CRPC: castration resistant prostate cancer
DepMap: The Cancer Dependency Map
ECDT: East Coast Dream Team
HPA: Human Protein Atlas
HR: Hazard Ratio
NEPC: neuroendocrine prostate cancer
OS: overall survival
PROMOTE: prostate cancer medically optimized genome‐enhanced therapy
SU2C/PCF: Stand Up 2 Cancer/Prostate Cancer Foundation
T2E: TMPRSS2‐ERG
UW/FHCRC: University of Washington/Fred Hutchinson Cancer Center
WCDT: West Coast Dream Team
WCM: Weill Cornell Medicine

High‐throughput methods for identifying novel therapeutic targets in cancer have been widely sought. Early work by the Cancer Cell Line Encyclopedia (CCLE) focused on screening drugs across hundreds of cancer cell lines [1]. However, drug screens cannot identify targets for which there is no compound yet. Large‐scale pooled functional genetic screens represent a way to simultaneously perturb thousands of genes and assess the effect on cell fitness. While CRISPR screens have been performed using various approaches in prostate cancer [2, 3, 4, 5, 6, 7], it is difficult to generalize their results given inconsistent methodology and small numbers of cell lines used. The Cancer Dependency Map (DepMap) has provided a more standardized and large‐scale screening of hundreds of cancer cell lines using both genome‐wide RNAi screens [8, 9], and subsequently CRISPR‐Cas9 (which will be abbreviated to just CRISPR subsequently) screens [10].

Prostate cancer is the most common malignancy in men according to the CDC US Cancer Statistics, with metastatic disease still lethal despite numerous FDA‐approved therapies. Utilization of functional screens [2, 3, 4, 5, 6, 7, 8, 9, 10] alone to identify prostate cancer dependencies and targets faces several challenges. Prostate cancer has only 10 total cell lines represented in DepMap [8, 9, 10], with an even smaller subset that is widely used, compared to other common cancer types with dozens of suitable model systems to represent the heterogeneity observed clinically. Therefore, it is critical to complement DepMap screen data [8, 9, 10] with other clinical data, especially in metastatic prostate cancer [11].

We assembled a dataset of 1012 metastatic prostate tumor samples with gene expression profiling [11]. This dataset was updated with additional overall survival (OS) data [12], to a new total of 525 patients, representing the largest aggregated clinical dataset in metastatic prostate cancer to our knowledge. Using these datasets, we first identified potentially druggable [13] targets based on *in vitro* genome‐wide functional screens [8, 9, 10]. We then examined clinical correlations and prioritized potential therapeutic targets in metastatic prostate cancer.

## Materials and Methods

### DepMap

DepMap data were downloaded from depmap.org. To do so, a model context was created with the following condition: Lineage is Prostate. Then, a custom download was initiated using this context as well as ‘Exclude columns and rows of NA's from download files’ and ‘Add cell line metadata to download’. CRISPR (DepMap Public 24Q2 + Score, Chronos), RNAi (Achilles + DRIVE + Marcotte, DEMETER2), and Batch corrected Expression Public 24Q4 data were then downloaded.

### Metastatic prostate cancer datasets

Gene expression data in metastatic prostate cancer were obtained from five published datasets: the University of Washington/Fred Hutchinson Cancer Center (UW/FHCRC) autopsy cohort (*n* = 254) [14, 15], a neuroendocrine prostate cancer (NEPC)‐enriched dataset from Weill Cornell Medicine (WCM) (*n* = 49) [16], the Castration Resistant Prostate Cancer (CRPC) datasets from the Stand Up 2 Cancer/Prostate Cancer Foundation (SU2C/PCF) East Coast Dream Team (ECDT; *n* = 328) [12, 17, 18], and West Coast Dream Team (WCDT; *n* = 240) [19, 20, 21, 22, 23, 24, 25] datasets. The Prostate Cancer Medically Optimized Genome‐Enhanced Therapy (PROMOTE) trial (*n* = 141) [26] contained pre‐ and postabiraterone exposure samples. Overall survival data were available for a subset of samples from the WCDT, ECDT, and PROMOTE cohorts (*n* = 525). Full details of these datasets are extensively described in their original publications [14, 15, 16, 17, 18, 19, 20, 21, 22, 23, 24, 25, 26]. DNA alteration calls were available for 780 samples, and adenocarcinoma versus NEPC status was available for 838 samples [11], as defined in each cohort.

### Bioinformatics analysis

We also limited our analysis to genes that are potentially targetable by existing molecules or clinical drugs (Tchem, Tclin respectively) according to Pharos (the web interface for the NIH Illuminating the Druggable Genome program) [27]. CRISPR screen data were used for prostate cancer and transformed prostate epithelial cell lines, but only RNAi screen data were available for NEPC. CRISPR and RNAi screen analysis was performed using DepMap [9]. Per the DepMap FAQ, a gene effect < −1 in CRISPR/RNAi screens is the median of all pan‐essential genes and a threshold that indicates strong killing. We identified genes where at least one prostate cancer/transformed cell line met this threshold in a cell line in which the gene was expressed, indicating some effect in at least a subset. Normalization of the gene expression data from the metastatic prostate cancer datasets was performed as previously described [11, 28, 29]. Pathway enrichment analysis was performed using the NIH david tool [30]. Protein expression was confirmed in the Human Protein Altas (HPA; www.proteinatlas.org).

### Statistical analysis

Survival analysis was performed using Cox regression with gene expression as a continuous variable, and overall survival as the endpoint in our samples with clinical outcomes. Multiple testing correction was performed using the Benjamini–Hochberg method. We also performed comparisons of expression between adenocarcinoma vs. NEPC and pre‐ vs. postabiraterone, and used a Wilcoxon signed‐rank test. All analysis was completed in r version 4.2.2 (R Foundation for Statistical Computing, Vienna, Austria).

## Results

### Overview

**Fig. 1:**
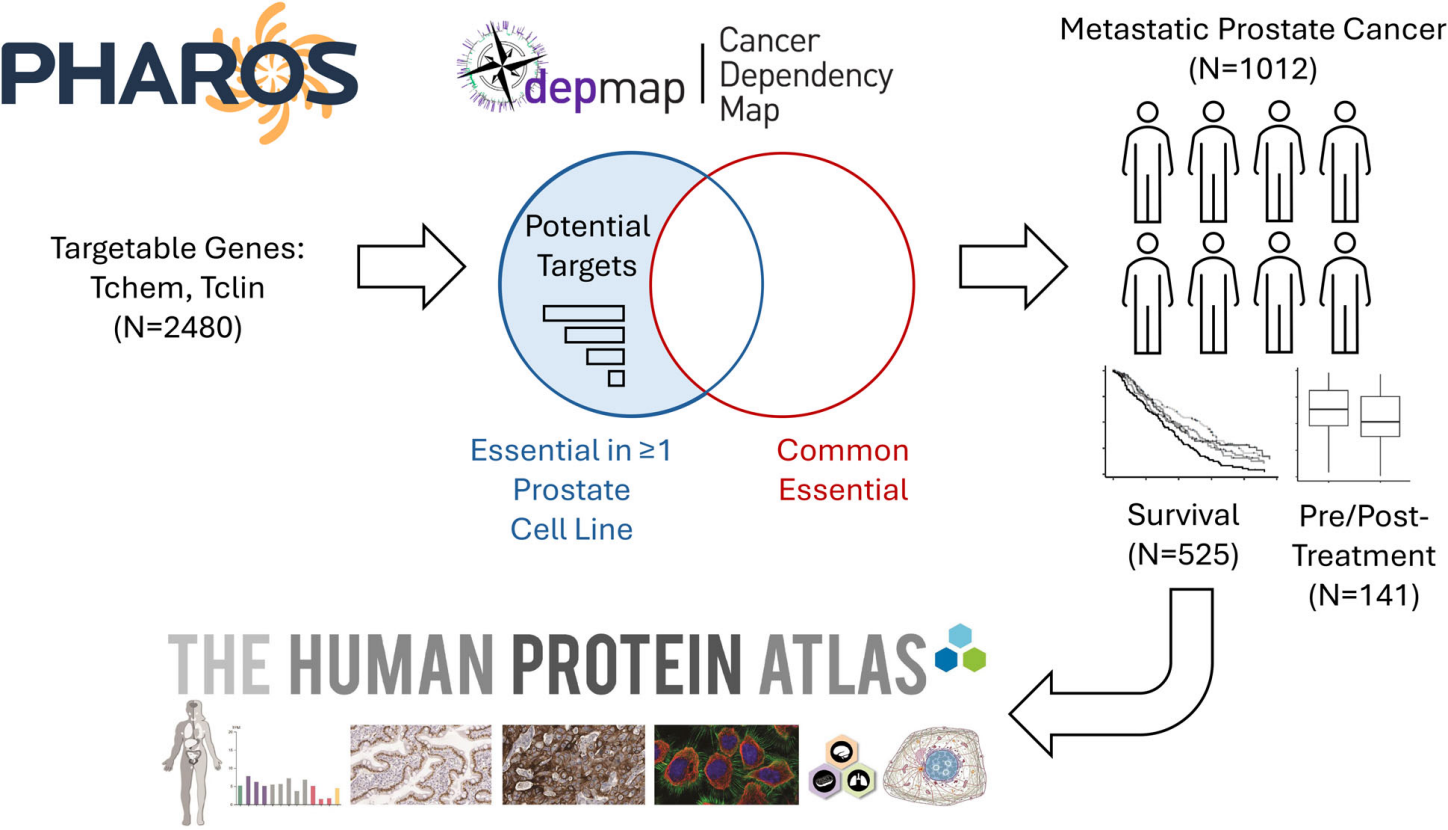
Schematic of this study. We limited our analysis to genes that are potentially targetable by existing molecules or clinical drugs (Tchem, Tclin respectively) according to Pharos (the web interface for the NIH Illuminating the Druggable Genome program). CRISPR/RNAi screen data from The Cancer Dependency Map (DepMap) were then used to identify targets that were essential for prostate cancer and transformed prostate epithelial cell lines but that were not commonly essential. Per the DepMap frequently asked questions (FAQ), a gene effect < −1 in CRISPR/RNAi screens is the median of all pan‐essential genes and a threshold that indicates strong killing. We identified genes where at least one prostate cancer/transformed cell line met this threshold, indicating some effect in at least a subset. We then identified genes where expression was associated with clinical outcomes from published datasets, resulting in nine genes including *AR*. Protein expression in the tumor was confirmed in the Human Protein Altas.

Prior studies indicate that 2480 genes are known to encode for proteins that bind to small molecules (PHAROS: Tchem), with some targeted by known drugs (PHAROS: Tclin). We queried DepMap CRISPR screens in prostate cancer cell lines as well as transformed prostate epithelial cell lines for evidence that these genes may alter cellular fitness. A gene effect < 0 indicates a deleterious effect, which can be observed for *AR* in VCaP (−0.333), LNCaP (−0.78), and 22RV1 (−1.28), all known to be androgen‐dependent [31]. On the other hand, known androgen‐independent cell lines [32, 33] (e.g., DU145, PC3) all had *AR* gene effects > 0. We therefore deemed genes where disruption caused a gene effect < −1 (the median of all pan‐essential genes indicating strong essentiality) as the most essential. Given the heterogeneity of prostate cancer and the small number of representative cell lines, we retained genes that were essential in one or more cell lines. NCIH660, the only neuroendocrine prostate cancer (NEPC) cell line, did not have CRISPR screen data available, but did have RNAi screen data, and so we utilized the RNAi screen data for this cell line in order to include NEPC, with the same thresholds. We also removed any common essential genes (per DepMap; Fig. 1).

### Functional screening reveals prostate cancer‐specific dependencies

**Fig. 2:**
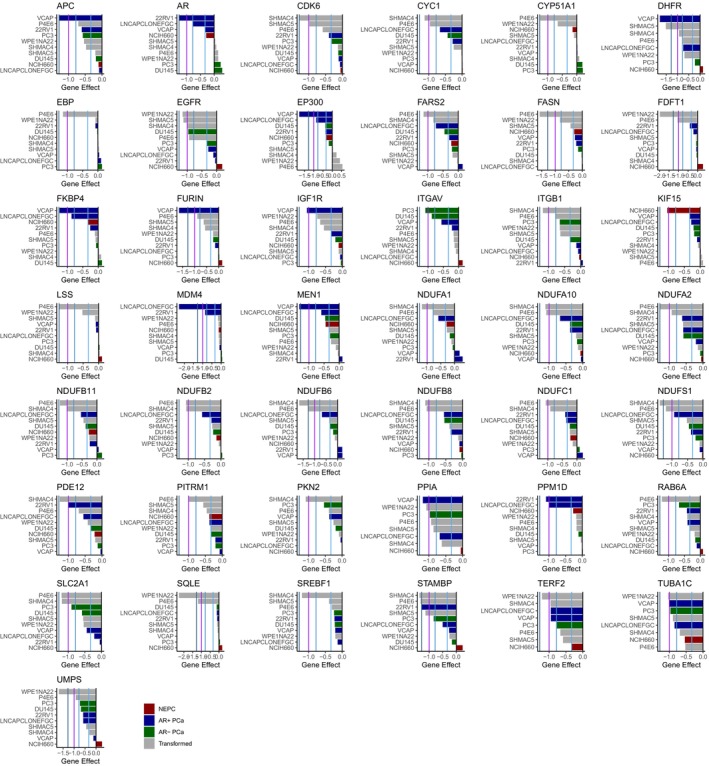
Essential prostate cancer gene dependencies. This figure shows the gene effects from The Cancer Dependency Map (DepMap) CRISPR/RNAi screens for all genes that are essential in at least one prostate cancer or transformed prostate cell line. This was defined as a gene effect of −1 (the median of all pan‐essential genes, and a threshold that indicates strong killing per the DepMap frequently asked questions (FAQ)) and indicated with the purple vertical line. The light/medium/dark blue vertical lines indicate the gene effect of AR CRISPR in all prostate cancer cell lines with a deleterious gene effect in the DepMap (−0.333: VCaP, −0.78: LNCaP, −1.28 in 22RV1).

**Fig. 3:**
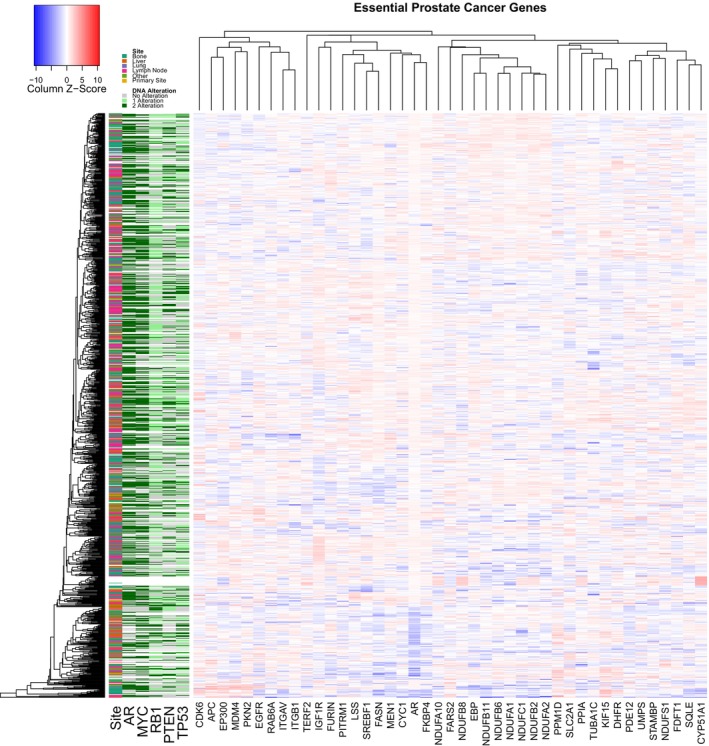
Expression of essential prostate cancer genes. Heatmap of the gene expression of the essential prostate cancer genes (in one or more cell lines) from Fig. 2, as well as the site of biopsy and key oncogenic and tumor suppressor alterations.

The integrated analysis of all prostate cancer cell line models resulted in 43 total genes with strong effects in prostate cancer/transformed cell lines from these functional screens (Fig. 2). Reassuringly, *AR* was identified in this list of potential therapeutic targets. Only one gene, *KIF15*, demonstrated a strong effect from disruption in the NEPC cell line. Using david pathway enrichment analysis [30], the prostate cancer dependencies were enriched for genes related to metabolism, and particularly cholesterol metabolism (Table S1). We next examined these targets in a previously assembled clinicogenomic metastatic prostate cancer dataset with 1012 metastatic prostate cancer samples [11]. In general, the gene expression did not show strong overall correlations with each other, biopsy site, or the most common genomic alterations in metastatic prostate cancer such as *AR* mutations/amplifications, *MYC* amplification, or *RB1/TP53/PTEN* loss (Fig. 3).

### Clinical outcomes associations

**Fig. 4:**
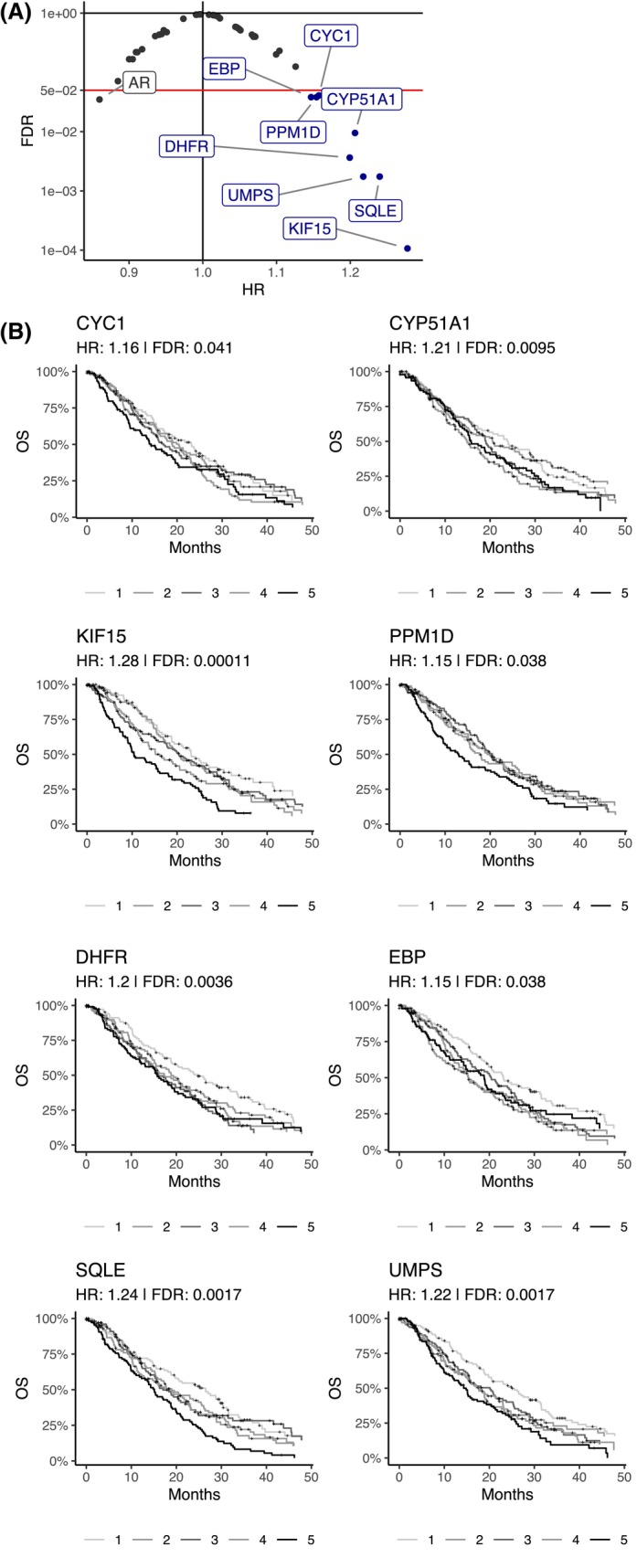
Clinical outcomes of essential prostate cancer genes. (A) Volcano plot of continuous gene expression Cox regression for Overall Survival (OS) of False Discover Rates (FDRs) and Hazard Ratios (HRs, scaled by standard deviation) of the essential prostate cancer genes (in one or more cell lines) from Figs 2 and 3. (B) Kaplan–Meier curves of the eight genes with an FDR < 0.05 and HR > 1, split into quintiles. The continuous HRs and FDRs are shown.

We next examined clinical outcomes by examining associations between genes with fitness effects *in vitro* and OS in the 525 samples where these data were available, the largest such dataset to our knowledge. Using continuous Cox regression, we found that 12 of the prostate cancer dependencies had statistically significant *P*‐values, which decreased to 9 after the Benjamini–Hochberg FDR correction was applied. Eight of these were associated with a Hazard Ratio (HR) > 1 indicating worse prognosis with increasing expression (Fig. 4). *AR* was the only gene where higher expression was associated with a better prognosis (compared to lower expression), which is concordant with the decrease in AR signaling in aggressive basal and NEPC tumors and the decreased efficacy of agents targeting AR [19, 28, 29]. The eight genes where higher expression was associated with worse prognosis (*CYC*, *CYP51A1*, *DHFR*, *EBP*, *KIF15*, *PPM1D*, *SQLE*, and *UMPS*) represent potential additional therapeutic targets in metastatic prostate cancer.

### NEPC, pre‐/postabiraterone

**Fig. 5:**
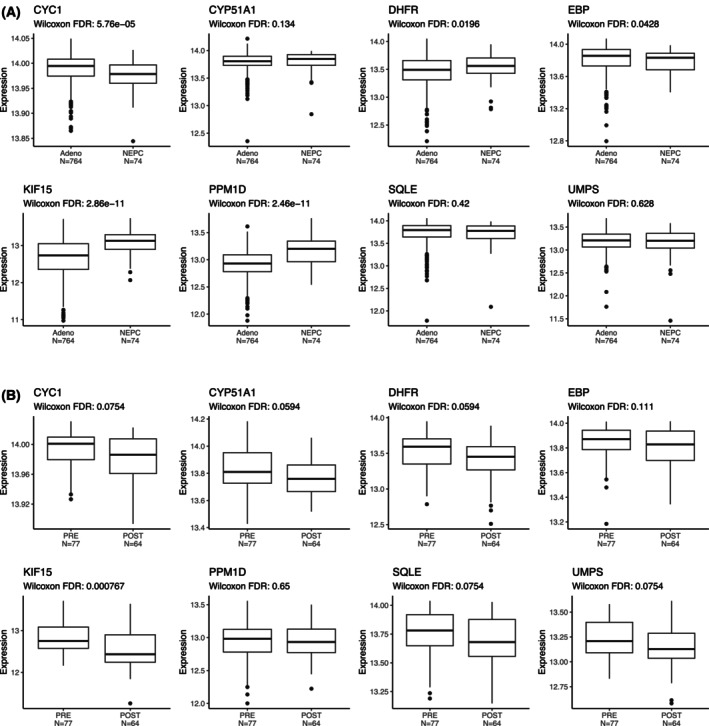
Histology, pre/post‐abiraterone expression of essential prostate cancer genes. Boxplots and Wilcoxon Rank‐Sum *P*‐values of expression (log‐transformed normalized gene rank) comparing (A) Adenocarcinoma vs. neuroendocrine prostate cancer (NEPC; as defined in the original publications of the cohorts) and (B) pre‐ vs. 12 weeks postabiraterone initiation samples in the Prostate Cancer Medically Optimized Genome‐Enhanced Therapy (PROMOTE) trial of the eight prognostic and essential prostate cancer genes (in one or more cell lines) from Fig. 4B.

NEPC is an aggressive, androgen‐independent subtype of prostate cancer [19, 28, 29]. We next determined whether these eight targets were differentially expressed in metastatic NEPC tumors compared to metastatic prostate adenocarcinomas. *DHFR*, *EBP*, *KIF15*, and *PPM1D* demonstrated higher expression in NEPC, whereas *CYC1* demonstrated lower expression (Fig. 5A). We also took advantage of the pretreatment samples before abiraterone therapy and those taken after 12 weeks of abiraterone from the PROMOTE trial to examine whether treatment with abiraterone modulated gene expression. Only *KIF15* was significantly, though only modestly, decreased from pretreatment to posttreatment (Fig. 5B), suggesting that these genes may remain targetable postabiraterone therapy.

### Protein expression

**Fig. 6:**
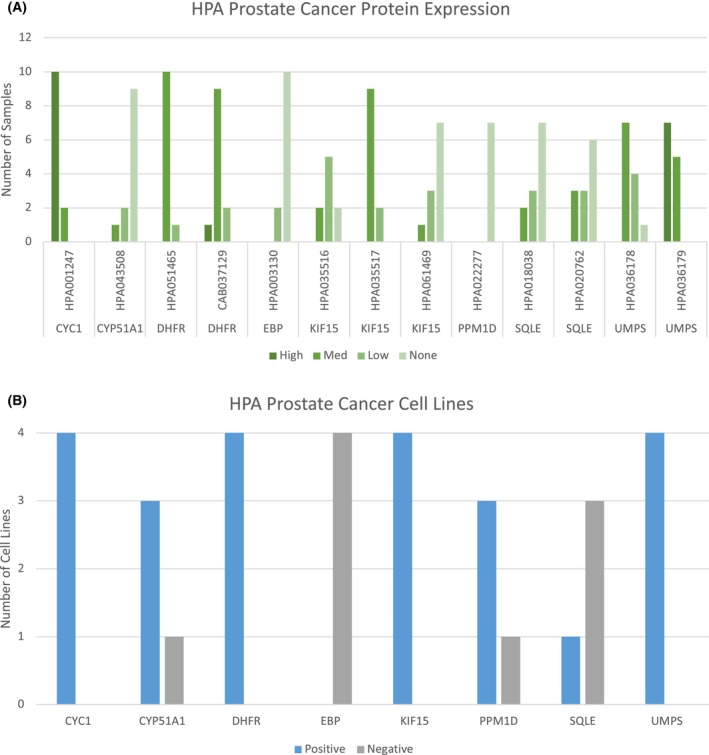
Human protein Atlas confirmation of tumor protein expression. (A) Barplots of Human Protein Atlas (HPA) expression in prostate cancer samples and (B) Barplots of HPA expression in prostate cancer cell lines (22Rv1, NCI‐H660, PC‐3, VCaP) of the eight genes from Figs 4B and 5. Positive/Negative defined per HPA based on detected/not detected using Mass Spectrometry.

Six of these eight genes demonstrated medium–high protein expression in one or more prostate tumors in the HPA (Fig. 6A). EBP did not show medium–high protein expression but did demonstrate low expression in two prostate tumors. While PPM1D did not demonstrate expression in clinical prostate tumors, it did show protein expression in 75% (3/4) prostate cancer cell lines tested (Fig. 6B). Altogether, all eight targets showed some evidence of protein expression in prostate cancers or cell lines. The HPA validates each of the antibodies for IHC and assigns them one of four classifications: Uncertain, Approved, Supported, and Enhanced. The antibodies for six of the eight targets were all Approved, Supported, or Enhanced. The antibodies used for DHFR and UMPS were classified as Uncertain. However, each of these proteins had two different antibodies each, providing orthogonal validation of protein expression in at least some prostate tumors.

## Discussion

Herein, we performed a clinicogenomic evaluation of genes conferring cell fitness defects *in vitro* and identified eight potential therapeutic targets in metastatic prostate cancer. Inactivation of these targets created strong phenotypes in one or more prostate cancer/transformed cell lines. Furthermore, increased gene expression of these targets was associated with worse prognosis in a large clinical cohort. There is also support for prostate cancer tissue or cell line protein expression of all eight targets, to varying degrees.

These eight genes have support as drug targets in prostate and other cancers. Notably, three genes involved in cholesterol synthesis pathways were identified, *CYP51A1*, *SQLE*, and *EBP*. Cholesterol synthesis is required by cancer cells to form membranes and supply energy for their rapid growth and division [34], which has led to exploration of its inhibition across multiple cancer types [35]. Of particular importance to prostate cancer, androgens are also synthesized from cholesterol precursors [36]. Androgen biosynthesis can play a role in prostate cancer progression and is a target of multiple drugs used to treat advanced disease: abiraterone [37] and ketaconazole [38]. While the primary target of these drugs is CYP17A1, both have also been shown to potentially target CYP51A1 [39, 40]. SQLE is upregulated across multiple cancers [41], both at the level of gene duplication and protein stabilization after p53/PTEN downregulation [35]. Elevated SQLE has also been associated with high‐risk prostate cancer, and inhibition promotes prostate cancer cell death [42, 43]. Its inhibition has also been shown to cause toxic accumulation of squalene in neuroendocrine lung cancer cells [44]. Interestingly, both *CYP51A1* and *SQLE* (squalene epoxidase) gene expression may be involved in suppressing the ferroptosis cell death pathway [45, 46]. Ferroptosis is triggered by accumulation of iron and lipid peroxidases, and inducers of ferroptosis have been proposed as promising cancer drugs in prostate and other cancers [47]. *EBP* (emopamil‐binding protein) encodes cholestenol delta‐isomerase, an endoplasmic reticulum membrane protein also involved in cholesterol biosynthesis that also likely binds and transports multiple drugs [48]. Inhibition of EBP has been shown to inhibit prostate cancer cell proliferation [49, 50].

We also identified genes needed for nucleotide synthesis, required for rapidly replicating cells. *DHFR* encodes the dihydrofolate reductase protein essential for synthesis of purines and thymidylate [51]. It is the target of the cancer therapeutic methotrexate as well as several newer drugs in clinical use and in development [51]. Methotrexate has been investigated in small trials in prostate cancer [52, 53], but only in combination with other chemotherapies. *UMPS* encodes uridine 5'‐monophosphate synthase, a bifunctional enzyme [54] involved in *de novo* synthesis of pyrimidines. Inhibitors of this and other steps of *de novo* pyrimidine synthesis are under active investigation in cancer [54, 55, 56, 57]. The pathway may make a promising drug target, as rapid proliferation of cancer cells depletes substrates from salvage pyrimidine biosynthesis pathways, increasing dependence on the *de novo* pathway [57]. Interestingly, this pathway has also been linked to cell defense against ferroptosis [54].

Other genes were identified which affect metabolic pathways, protein transport, and apoptosis. The *CYC1* gene encodes cytochrome C1, a mitochondrial protein that is part of the electron transport chain driving oxidative phosphorylation, which may play a role in metabolic plasticity that promotes stemness, drug resistance, and metastasis [58]. This metabolic plasticity is increasingly recognized in prostate cancer [58], though the role of CYC1 specifically in this process has not been explored. However, elevated CYC1 with a potential function in resisting apoptosis has been found in uveal melanoma [59] and osteosarcoma [60, 61]. Kinesin family member 15 (KIF15) is overexpressed in multiple cancers and is involved in mitotic spindle assembly and protein transport [62]. Inhibitors of KIF15 are under development and have been demonstrated to prevent mitosis preclinically in combination with other drugs [63]. Its role in multiple cancers is currently being explored [64, 65, 66, 67], including prostate cancer, in which it may play a role in resistance to AR inhibition [68, 69]. The PPM1D gene encodes protein phosphatase, Mg2+/Mn2+‐dependent 1D, a Ser/Thr protein phosphatase also known as wild‐type p53‐induced phosphatase 1 (Wip1) and is a negative regulator of p53‐induced apoptosis. Germline mutations in this gene and overexpression are associated with increased risks of multiple cancers [70], including prostate cancer [70, 71, 72] and many hematological malignancies [73].

High‐throughput CRISPR and RNAi screens have several limitations, and validation of anticancer activity can depend on the model system, method of inhibition, and other factors. Even in the setting of known driver alterations such as the TMPRSS2‐ERG (T2E) fusion, there can be inconsistent effects. VCAP contains a T2E fusion and is also highly dependent on ERG, with a gene effect of −1.43 for ERG in the DepMap CRISPR screen. NCIH660 also has a T2E fusion but with no gene effect for ERG in the DepMap CRISPR screen, which may be because NCIH660 is an NEPC model and thus likely has other alterations driving this transformation that make it less dependent on the T2E fusion. Nonetheless, these screens identify AR as a key target in prostate cancer. The other eight targets are associated with worse clinical outcomes and demonstrate varying levels of literature support as potential targets, supporting further preclinical and clinical investigations. However, it is important to note that these targets are only essential in a subset of cell lines. Given the differences molecularly between the cell lines, further investigation into potential interactions with specific DNA alterations is important to better understand the potentially targetable patient subgroups. Furthermore, additional protein studies will be important, given that HPA focuses on localized disease and the antibodies used have varying levels of validation.

## Conflict of interest

SGZ has patent applications with Veracyte on molecular signatures in prostate cancer unrelated to this work, and a family member employed by Artera, and with stock in Exact Sciences. MSj reports speaker fees from Astellas and consulting fees and Advisory Board for Astellas/Adelphi Targis, unrelated to this work. PSN has served as a paid consultant to Janssen, Genentech, Pfizer, and AstraZeneca and received research support from Janssen for work unrelated to the present report.

## Author contributions

MS and SGZ conceived and designed the project. WC, MZ, DQ, MS, HL, IC, PSN, MS, and SGZ acquired the data. EF, EF, ISN, LGN, WC, MZ, DQ, MS, HL, PSN, MS, and SGZ analyzed and interpreted the data. TB, MS, and SGZ wrote the paper.

## Supporting information


**Table S1.** Pathway analysis.

## Data Availability

UW/FHCRC, WCM, and the ECDT data are available on cBioPortal. The PROMOTE cohort data are available on dbGaP (phs001141). The WCDT were obtained from publication supplemental materials as well as dbGaP (phs001648) and EGA (EGAD00001008991, EGAD00001008487, EGAD00001009065, EGAS00001006649).

## References

[1] Barretina J , Caponigro G , Stransky N , Venkatesan K , Margolin AA , Kim S , Wilson CJ , Lehár J , Kryukov GV , Sonkin D *et al*. (2012) The cancer cell line encyclopedia enables predictive modelling of anticancer drug sensitivity. Nature 483, 603–607. 22460905 10.1038/nature11003 PMC3320027

[2] Ahmed M , Soares F , Xia J‐H , Yang Y , Li J , Guo H , Su P , Tian Y , Lee HJ , Wang M *et al*. (2021) CRISPRi screens reveal a DNA methylation‐mediated 3D genome dependent causal mechanism in prostate cancer. Nat Commun 12, 1781. 33741908 10.1038/s41467-021-21867-0 PMC7979745

[3] Das R , Sjöström M , Shrestha R , Yogodzinski C , Egusa EA , Chesner LN , Chen WS , Chou J , Dang DK , Swinderman JT *et al*. (2021) An integrated functional and clinical genomics approach reveals genes driving aggressive metastatic prostate cancer. Nat Commun 12, 4601. 34326322 10.1038/s41467-021-24919-7 PMC8322386

[4] Rodriguez Y , Unno K , Mihai T I , Chalmers ZR , Yoo YA , Vatapalli R , Sagar V , Yu J , Lysy B , Hussain M *et al*. (2022) A genome‐wide CRISPR activation screen identifies PRRX2 as a regulator of enzalutamide resistance in prostate cancer. Cancer Res 82, 2110–2123. 35405009 10.1158/0008-5472.CAN-21-3565 PMC9177667

[5] Tang S , Sethunath V , Metaferia N , Nogueira M , Gallant D , Garner E , Lairson L , Penney C , Li J , Gelbard M *et al*. (2022) A genome‐scale CRISPR screen reveals PRMT1 as a critical regulator of androgen receptor signaling in prostate cancer. Cell Rep 38, 110417. 35196489 10.1016/j.celrep.2022.110417 PMC9036938

[6] Tsujino T , Takai T , Hinohara K , Gui F , Tsutsumi T , Bai X , Miao C , Feng C , Gui B , Sztupinszki Z *et al*. (2023) CRISPR screens reveal genetic determinants of PARP inhibitor sensitivity and resistance in prostate cancer. Nat Commun 14, 252. 36650183 10.1038/s41467-023-35880-y PMC9845315

[7] Fei T , Chen Y , Xiao T , Li W , Cato L , Zhang P , Cotter M , Bowden M , Lis R , Zhao S *et al*. (2017) Genome‐wide CRISPR screen identifies HNRNPL as a prostate cancer dependency regulating RNA splicing. Proc Natl Acad Sci U S A 114, E5207–E5215. 28611215 10.1073/pnas.1617467114 PMC5495225

[8] McDonald E , de Weck A , Schlabach MR , Billy E , Mavrakis KJ , Hoffman GR , Belur D , Castelletti D , Frias E , Gampa K *et al*. (2017) Project DRIVE: a compendium of cancer dependencies and synthetic lethal relationships uncovered by large‐scale, deep RNAi screening. Cell 170, 577–592 e510. 28753431 10.1016/j.cell.2017.07.005

[9] Tsherniak A , Vazquez F , Montgomery P , Weir B , Kryukov G , Cowley G , Gill S , Harrington W , Pantel S , Krill‐Burger J *et al*. (2017) Defining a cancer dependency map. Cell 170, 564–576 e516. 28753430 10.1016/j.cell.2017.06.010 PMC5667678

[10] Dempster JM , Pacini C , Pantel S , Behan F , Green T , Krill‐Burger J , Beaver C , Younger S , Zhivich V , Najgebauer H *et al*. (2019) Agreement between two large pan‐cancer CRISPR‐Cas9 gene dependency data sets. Nat Commun 10, 5817. 31862961 10.1038/s41467-019-13805-y PMC6925302

[11] Sharifi MN , Feng E , Rydzewski N , Taylor A , Sperger J , Shi Y , Helzer K , Bootsma M , Carreno V , Chang A *et al*. (2025) Adverse prognosis gene expression patterns in metastatic castration‐resistant prostate cancer. Mol Oncol doi: 10.1002/1878-0261.70001 PMC12330944 39985777

[12] Graham LS , Haffner M , Sayar E , Gawne A , Schweizer M , Pritchard C , Coleman I , Nelson P and Yu E (2023) Clinical, pathologic, and molecular features of amphicrine prostate cancer. Prostate 83, 641–648. 36779357 10.1002/pros.24497 PMC11023623

[13] Kelleher KJ , Sheils T , Mathias S , Yang J , Metzger V , Siramshetty V , Nguyen DT , Jensen L , Vidović D , Schürer S *et al*. (2023) Pharos 2023: an integrated resource for the understudied human proteome. Nucleic Acids Res 51, D1405–D1416. 36624666 10.1093/nar/gkac1033 PMC9825581

[14] Kumar A , Coleman I , Morrissey C , Zhang X , True LD , Gulati R , Etzioni R , Bolouri H , Montgomery B , White T *et al*. (2016) Substantial interindividual and limited intraindividual genomic diversity among tumors from men with metastatic prostate cancer. Nat Med 22, 369–378. 26928463 10.1038/nm.4053 PMC5045679

[15] Labrecque MP , Coleman IM , Brown LG , True LD , Kollath L , Lakely B , Nguyen HM , Yang YC , da Costa RMG , Kaipainen A *et al*. (2019) Molecular profiling stratifies diverse phenotypes of treatment‐refractory metastatic castration‐resistant prostate cancer. J Clin Invest 129, 4492–4505. 31361600 10.1172/JCI128212 PMC6763249

[16] Beltran H , Prandi D , Mosquera JM , Benelli M , Puca L , Cyrta J , Marotz C , Giannopoulou E , Chakravarthi BV , Varambally S *et al*. (2016) Divergent clonal evolution of castration‐resistant neuroendocrine prostate cancer. Nat Med 22, 298–305. 26855148 10.1038/nm.4045 PMC4777652

[17] Abida W , Cyrta J , Heller G , Prandi D , Armenia J , Coleman I , Cieslik M , Benelli M , Robinson D , Van Allen EM *et al*. (2019) Genomic correlates of clinical outcome in advanced prostate cancer. Proc Natl Acad Sci U S A 116, 11428–11436. 31061129 10.1073/pnas.1902651116 PMC6561293

[18] Robinson D , Van Allen EM , Wu Y‐M , Schultz N , Lonigro RJ , Mosquera J‐M , Montgomery B , Taplin M‐E , Pritchard CC , Attard G *et al*. (2015) Integrative clinical genomics of advanced prostate cancer. Cell 161, 1215–1228. 26000489 10.1016/j.cell.2015.05.001 PMC4484602

[19] Zhao SG , Chen W , Li H , Foye A , Zhang M , Sjöström M , Aggarwal R , Playdle D , Liao A , Alumkal J *et al*. (2020) The DNA methylation landscape of advanced prostate cancer. Nat Genet 52, 778–789. 32661416 10.1038/s41588-020-0648-8 PMC7454228

[20] Alumkal JJ , Sun D , Lu E , Beer TM , Thomas GV , Latour E , Aggarwal R , Cetnar J , Ryan CJ , Tabatabaei S *et al*. (2020) Transcriptional profiling identifies an androgen receptor activity‐low, stemness program associated with enzalutamide resistance. Proc Natl Acad Sci U S A 117, 12315–12323. 32424106 10.1073/pnas.1922207117 PMC7275746

[21] Aggarwal R , Huang J , Alumkal JJ , Zhang L , Feng FY , Thomas GV , Weinstein AS , Friedl V , Zhang C , Witte ON *et al*. (2018) Clinical and genomic characterization of treatment‐emergent small‐cell neuroendocrine prostate cancer: a multi‐institutional prospective study. J Clin Oncol 36, 2492–2503. 29985747 10.1200/JCO.2017.77.6880 PMC6366813

[22] Lundberg A , Zhang M , Aggarwal R , Li H , Zhang L , Foye A , Sjöström M , Chou J , Chang K , Moreno‐Rodriguez T *et al*. (2023) The genomic and Epigenomic landscape of double‐negative metastatic prostate cancer. Cancer Res 83, 2763–2774. 37289025 10.1158/0008-5472.CAN-23-0593 PMC10425725

[23] Quigley DA , Dang HX , Zhao SG , Lloyd P , Aggarwal R , Alumkal JJ , Foye A , Kothari V , Perry MD , Bailey AM *et al*. (2018) Genomic hallmarks and structural variation in metastatic prostate cancer. Cell 174, 758–769 e759. 30033370 10.1016/j.cell.2018.06.039 PMC6425931

[24] Westbrook TC , Guan X , Rodansky E , Flores D , Liu CJ , Udager AM , Patel RA , Haffner MC , Hu Y‐M , Sun D *et al*. (2022) Transcriptional profiling of matched patient biopsies clarifies molecular determinants of enzalutamide‐induced lineage plasticity. Nat Commun 13, 5345. 36109521 10.1038/s41467-022-32701-6 PMC9477876

[25] Chen WS , Aggarwal R , Zhang L , Zhao SG , Thomas GV , Beer TM , Quigley DA , Foye A , Playdle D , Huang J *et al*. (2019) Genomic drivers of poor prognosis and enzalutamide resistance in metastatic castration‐resistant prostate cancer. Eur Urol 76, 562–571. 30928160 10.1016/j.eururo.2019.03.020 PMC6764911

[26] Sicotte H , Kalari KR , Qin S , Dehm SM , Bhargava V , Gormley M , Tan W , Sinnwell JP , Hillman DW , Li Y *et al*. (2022) Molecular profile changes in patients with castrate‐resistant prostate cancer pre‐ and post‐Abiraterone/prednisone treatment. Mol Cancer Res 20, 1739–1750. 36135372 10.1158/1541-7786.MCR-22-0099 PMC9716248

[27] Sheils T , Mathias SL , Siramshetty VB , Bocci G , Bologa CG , Yang JJ , Waller A , Southall N , Nguyen DT and Oprea TI (2020) How to illuminate the Druggable genome using Pharos. Curr Protoc Bioinformatics 69, e92. 31898878 10.1002/cpbi.92 PMC7818358

[28] Aggarwal R , Rydzewski N , Zhang L , Foye A , Kim W , Helzer K , Bakhtiar H , Chang S , Perry M , Gleave M *et al*. (2021) Prognosis associated with luminal and basal subtypes of metastatic prostate cancer. JAMA Oncol 7, 1644–1652. doi: 10.1001/jamaoncol.2021.3987 34554200 PMC8461554

[29] Feng E , Rydzewski NR , Zhang M , Lundberg A , Bootsma M , Helzer KT , Lang JM , Aggarwal R , Small EJ , Quigley DA *et al*. (2022) Intrinsic molecular subtypes of metastatic castration‐resistant prostate cancer. Clin Cancer Res 28, 5396–5404. 36260524 10.1158/1078-0432.CCR-22-2567 PMC9890931

[30] Huang da W , Sherman BT and Lempicki RA (2009) Systematic and integrative analysis of large gene lists using DAVID bioinformatics resources. Nat Protoc 4, 44–57. 19131956 10.1038/nprot.2008.211

[31] Sobel RE and Sadar MD (2005) Cell lines used in prostate cancer research: a compendium of old and new lines—part 1. J Urol 173, 342–359. 15643172 10.1097/01.ju.0000141580.30910.57

[32] Diaz M , Karlsson S , Szekeres F , Faresjö M , Lund D and Larsson D (2021) Differential expression of protein disulfide‐isomerase A3 isoforms, PDIA3 and PDIA3N, in human prostate cancer cell lines representing different stages of prostate cancer. Mol Biol Rep 48, 2429–2436. 33761087 10.1007/s11033-021-06277-1 PMC8060222

[33] Opoku‐Acheampong AB , Nelsen MK , Unis D and Lindshield BL (2012) The effect of finasteride and dutasteride on the growth of WPE1‐NA22 prostate cancer xenografts in nude mice. PLoS One 7, e29068. 22242155 10.1371/journal.pone.0029068 PMC3252297

[34] Gu Q , Wang Y , Yi P and Cheng C (2024) Theoretical framework and emerging challenges of lipid metabolism in cancer. Semin Cancer Biol 108, 48–70. 39674303 10.1016/j.semcancer.2024.12.002

[35] Shangguan X , Ma Z , Yu M , Ding J , Xue W and Qi J (2022) Squalene epoxidase metabolic dependency is a targetable vulnerability in castration‐resistant prostate cancer. Cancer Res 82, 3032–3044. 35767703 10.1158/0008-5472.CAN-21-3822

[36] Miller WL (2017) Steroidogenesis: unanswered questions. Trends Endocrinol Metab 28, 771–793. 29031608 10.1016/j.tem.2017.09.002

[37] De JS , Logothetis CJ , Molina A , Fizazi K , North S , Chu L , Chi KN , Jones RJ , Goodman , Saad F *et al*. (2011) Abiraterone and increased survival in metastatic prostate cancer. N Engl J Med 364, 1995–2005. 21612468 10.1056/NEJMoa1014618 PMC3471149

[38] Patel V , Liaw B and Oh W (2018) The role of ketoconazole in current prostate cancer care. Nat Rev Urol 15, 643–651. 30154429 10.1038/s41585-018-0077-y

[39] Masamrekh R , Kuzikov A , Veselovsky A , Toropygin I , Shkel T , Strushkevich N , Gilep A , Usanov S , Archakov A and Shumyantseva V (2018) Interaction of 17alpha‐hydroxylase, 17(20)‐lyase (CYP17A1) inhibitors – abiraterone and galeterone – with human sterol 14alpha‐demethylase (CYP51A1). J Inorg Biochem 186, 24–33. 29807244 10.1016/j.jinorgbio.2018.05.010

[40] Strushkevich N , Usanov SA and Park HW (2010) Structural basis of human CYP51 inhibition by antifungal azoles. J Mol Biol 397, 1067–1078. 20149798 10.1016/j.jmb.2010.01.075

[41] Cirmena G , Franceschelli P , Isnaldi E , Ferrando L , De M , Ballestrero A and Zoppoli G (2018) Squalene epoxidase as a promising metabolic target in cancer treatment. Cancer Lett 425, 13–20. 29596888 10.1016/j.canlet.2018.03.034

[42] Stopsack KH , Gerke T , Sinnott J , Penney K , Tyekucheva S , Sesso H , Andersson SO , Andrén O , Cerhan J , Giovannucci E *et al*. (2016) Cholesterol metabolism and prostate cancer lethality. Cancer Res 76, 4785–4790. 27325648 10.1158/0008-5472.CAN-16-0903 PMC4987257

[43] Zhao SG , Evans J , Kothari V , Sun G , Larm A , Mondine V , Schaeffer E , Ross A , Klein E , den R *et al*. (2016) The landscape of prognostic outlier genes in high‐risk prostate cancer. Clin Cancer Res 22, 1777–1786. 26631616 10.1158/1078-0432.CCR-15-1250

[44] Mahoney CE , Pirman D , Chubukov V , Sleger T , Hayes S , Fan Z , Allen E , Chen Y , Huang L , Liu M *et al*. (2019) A chemical biology screen identifies a vulnerability of neuroendocrine cancer cells to SQLE inhibition. Nat Commun 10, 96. 30626880 10.1038/s41467-018-07959-4 PMC6327044

[45] Li Y , Ran Q , Duan Q , Jin J , Wang Y , Yu L , Wang C , Zhu Z , Chen X , Weng L *et al*. (2024) 7‐Dehydrocholesterol dictates ferroptosis sensitivity. Nature 626, 411–418. 38297130 10.1038/s41586-023-06983-9 PMC11298758

[46] Zhang R , Zhang L , Fan S , Wang L , Wang B and Wang L (2024) Squalene monooxygenase (SQLE) protects ovarian cancer cells from ferroptosis. Sci Rep 14, 22646. 39349544 10.1038/s41598-024-72506-9 PMC11442994

[47] Liang J , Liao Y , Wang P , Yang K , Wang Y , Wang K , Zhong B , Zhou D , Cao Q , Li J *et al*. (2023) Ferroptosis landscape in prostate cancer from molecular and metabolic perspective. Cell Death Dis 9, 128. 10.1038/s41420-023-01430-0 PMC10105735 37061523

[48] Long T , Hassan A , Thompson B , McDonald J , Wang J and Li X (2019) Structural basis for human sterol isomerase in cholesterol biosynthesis and multidrug recognition. Nat Commun 10, 2452. 31165728 10.1038/s41467-019-10279-w PMC6549186

[49] Berardi F , Abate C , Ferorelli S , de A , Leopoldo M , Colabufo N , Niso M and Perrone R (2008) Novel 4‐(4‐aryl)cyclohexyl‐1‐(2‐pyridyl)piperazines as Delta(8)‐Delta(7) sterol isomerase (emopamil binding protein) selective ligands with antiproliferative activity. J Med Chem 51, 7523–7531. 19053780 10.1021/jm800965b

[50] Berthois Y , Bourrié B , Galiègue S , Vidal H , Carayon P , Martin P and Casellas P (2003) SR31747A is a sigma receptor ligand exhibiting antitumoural activity both in vitro and in vivo. Br J Cancer 88, 438–446. 12569389 10.1038/sj.bjc.6600709 PMC2747535

[51] Raimondi MV , Randazzo O , la M , Barone G , Vignoni E , Rossi D and Collina S (2019) DHFR inhibitors: Reading the past for discovering novel anticancer agents. Molecules 24, 1140. doi: 10.3390/molecules24061140 30909399 PMC6471984

[52] Wozniak AJ , Blumenstein BA , Crawford ED , Boileau M , Rivkin SE and Fletcher WS (1993) Cyclophosphamide, methotrexate, and 5‐fluorouracil in the treatment of metastatic prostate cancer. A Southwest Oncology Group Study. Cancer 71, 3975–3978. 8508363 10.1002/1097-0142(19930615)71:12<3975::aid-cncr2820711229>3.0.co;2-d

[53] Loening SA , Beckle S , Brady MF , Chu TM , Dekernion JB , Dhabuwala C , Gaeta JF , Gibbons RP , Mckiel CF , McLeod DG *et al*. (1983) Comparison of estramustine phosphate, methotrexate and cis‐platinum in patients with advanced, hormone refractory prostate cancer. J Urol 129, 1001–1006. 6343629 10.1016/s0022-5347(17)52509-4

[54] Yang C , Zhao Y , Wang L , Guo Z , Ma L , Yang R , Wu Y , Li X , Niu J , Chu Q *et al*. (2023) De novo pyrimidine biosynthetic complexes support cancer cell proliferation and ferroptosis defence. Nat Cell Biol 25, 836–847. 37291265 10.1038/s41556-023-01146-4

[55] Lafita‐Navarro MC , Venkateswaran N , Kilgore J , Kanji S , Han J , Barnes S , Williams N , Buszczak M , Burma S and Conacci‐Sorrell M (2020) Inhibition of the de novo pyrimidine biosynthesis pathway limits ribosomal RNA transcription causing nucleolar stress in glioblastoma cells. PLoS Genet 16, e1009117. 33201894 10.1371/journal.pgen.1009117 PMC7707548

[56] Wittmann JG , Heinrich D , Gasow K , Frey A , Diederichsen U and Rudolph M (2008) Structures of the human orotidine‐5'‐monophosphate decarboxylase support a covalent mechanism and provide a framework for drug design. Structure 16, 82–92. 18184586 10.1016/j.str.2007.10.020

[57] Pal S , Kaplan J , Nguyen H , Stopka S , Savani M , Regan M , Nguyen QD , Jones K , Moreau L , Peng J *et al*. (2022) A druggable addiction to de novo pyrimidine biosynthesis in diffuse midline glioma. Cancer Cell 40, 957–972.e910. 35985342 10.1016/j.ccell.2022.07.012 PMC9575661

[58] Fontana F , Anselmi M and Limonta P (2023) Unraveling the peculiar features of mitochondrial metabolism and dynamics in prostate cancer. Cancers (Basel) 15, 1192. 36831534 10.3390/cancers15041192 PMC9953833

[59] Zhan Z , Lin K and Wang T (2024) Construction of oxidative phosphorylation‐related prognostic risk score model in uveal melanoma. BMC Ophthalmol 24, 204. 38698303 10.1186/s12886-024-03441-6 PMC11067154

[60] Li G , Fu D , Liang W , Fan L , Chen K , Shan L , Hu S , Ma X , Zhou K and Cheng B (2014) CYC1 silencing sensitizes osteosarcoma cells to TRAIL‐induced apoptosis. Cell Physiol Biochem 34, 2070–2080. 25562155 10.1159/000366402

[61] Li G , Zhang W , Zeng H , Chen L , Wang W , Liu J , Zhang Z and Cai Z (2009) An integrative multi‐platform analysis for discovering biomarkers of osteosarcoma. BMC Cancer 9, 150. 19445706 10.1186/1471-2407-9-150 PMC2691408

[62] Mi J , Ma S , Chen W , Kang M , Xu M , Liu C , Li B , Wu F , Liu F , Zhang Y *et al*. (2022) Integrative pan‐cancer analysis of KIF15 reveals its diagnosis and prognosis value in nasopharyngeal carcinoma. Front Oncol 12, 772816. 35359374 10.3389/fonc.2022.772816 PMC8963360

[63] Solon AL , Zaniewski T , O'Brien P , Clasby M , Hancock W and Ohi R (2022) Synergy between inhibitors of two mitotic spindle assembly motors undermines an adaptive response. Mol Biol Cell 33, ar132. 36200902 10.1091/mbc.E22-06-0225 PMC9727797

[64] Wang J , Wang D , Fei Z , Feng D , Zhang B , Gao P , Hu G , Li W , Huang X , Chen D *et al*. (2021) KIF15 knockdown suppresses gallbladder cancer development. Eur J Cell Biol 100, 151182. 34781077 10.1016/j.ejcb.2021.151182

[65] Xie W , Zhang L , Shen J , Lai F , Han W and Liu X (2024) Knockdown of CENPM activates cGAS‐STING pathway to inhibit ovarian cancer by promoting pyroptosis. BMC Cancer 24, 551. 38693472 10.1186/s12885-024-12296-5 PMC11064423

[66] Guo Q , Qiu P , Pan K , Liang H , Liu Z and Lin J (2024) Integrated machine learning algorithms identify KIF15 as a potential prognostic biomarker and correlated with stemness in triple‐negative breast cancer. Sci Rep 14, 21449. 39271768 10.1038/s41598-024-72406-y PMC11399402

[67] Yu W , Han S , Hu S , Ru L , Hua C , Xue G , Zhang G , Lv K , Ge H , Wang M *et al*. (2024) KIF15 promotes human glioblastoma progression under the synergistic transactivation of REST and P300. Int J Biol Sci 20, 5127–5144. 39430242 10.7150/ijbs.98668 PMC11488581

[68] Gao L , Zhang W , Zhang J , Liu J , Sun F , Liu H , Hu J , Wang X , Wang X , Su P *et al*. (2021) KIF15‐mediated stabilization of AR and AR‐V7 contributes to enzalutamide resistance in prostate cancer. Cancer Res 81, 1026–1039. 33277366 10.1158/0008-5472.CAN-20-1965

[69] Gao L , Zhao R , Liu J , Zhang W , Sun F , Yin Q , Wang X , Wang M , Feng T , Qin Y *et al*. (2021) KIF15 promotes progression of castration resistant prostate cancer by activating EGFR signaling pathway. Front Oncol 11, 679173. 34804913 10.3389/fonc.2021.679173 PMC8599584

[70] Tsoulos N , Agiannitopoulos K , Potska K , Katseli A , Ntogka C , Pepe G , Bouzarelou D , Papathanasiou A , Grigoriadis D , Tsaousis GN *et al*. (2024) The clinical and genetic landscape of hereditary cancer: experience from a single clinical diagnostic laboratory. Cancer Genomics Proteomics 21, 448–463. 39191493 10.21873/cgp.20463 PMC11363926

[71] Cardoso M , Paulo P , Maia S and Teixeira MR (2016) Truncating and missense PPM1D mutations in early‐onset and/or familial/hereditary prostate cancer patients. Genes Chromosomes Cancer 55, 954–961. 27401275 10.1002/gcc.22393

[72] Tang T , Tan X , Wang Z , Wang S , Wang Y , Xu J , Wei X , Zhang D , Liu Q , Jiang J *et al*. (2022) Germline mutations in patients with early‐onset prostate cancer. Front Oncol 12, 826778. 35734583 10.3389/fonc.2022.826778 PMC9207501

[73] Husby S , Hjermind Justesen E and Grønbæk K (2021) Protein phosphatase, Mg(2+)/Mn(2+)‐dependent 1D (PPM1D) mutations in haematological cancer. Br J Haematol 192, 697–705. 33616916 10.1111/bjh.17120

